# Associations between Autistic-like Traits and Imagery Ability

**DOI:** 10.3390/vision8010013

**Published:** 2024-03-12

**Authors:** Takao Hatakeyama

**Affiliations:** Independent Researcher, Yamagata 990-8560, Japan; t_hatakeyama@cc.yamagata-u.ac.jp

**Keywords:** autism spectrum disorder (ASD), Autism Spectrum Quotient (AQ), imagery ability, vividness, imagery type, college students, Vividness of Visual Imagery Questionnaire (VVIQ), Questionnaire upon Mental Imagery (QMI), Verbalizer–Visualizer Questionnaire (VVQ)

## Abstract

This article examines empirical associations between qualities of the imagination, mental imagery, and cognitive abilities with special reference to autism. This study is the first to explore the empirical relationships between autistic-like traits and tests of imagery differences. Imaginative impairments and distinctive sensory characteristics in individuals with autism spectrum disorder (ASD) should be reflected in their interactions with mental imagery. However, the relationship between ASD and imaging traits remains unclear. Based on the hypothesis that the degree of autistic-like traits is reflected in imagery traits, this study examined how the individual Autism Spectrum Quotient (AQ) relates to imagery ability in 250 college students. Two vividness tests and one imagery-type test were used to assess imagery ability. Scores in each imagery test were compared between the high-scoring group classified by the AQ and the rest of the participants and between the low-scoring group classified by the AQ and the other participants. This study also directly compared imagery test scores between the high- and low-scoring groups. In terms of the total AQ score, the high-scoring group exhibited lower visualization scores. Regarding AQ subscales, “imagination” had the most extensive relationship with imagery traits, with the high-scoring group (unimaginative) showing lower imagery vividness across various modalities as well as lower visualization and verbalization scores. This was followed by the “attention to detail” subscale, on which the high-scoring group (attentive to detail) showed higher vividness of visual imagery. The results of the low-scoring group exhibited, on the whole, opposite imagery tendencies to the high-scoring group. The results indicate that autistic-like traits are associated with qualities of the imagination and especially mental imagery ability.

## 1. Introduction

People image the world according to their individual neurocognitive traits and abilities. Here, we examine how imagery and imagination differences are associated with traits of autism spectrum disorder (ASD). ASD is characterized by persistent deficits in social communication and social interaction (e.g., deficits in non-verbal communicative behaviors and difficulties in imaginative play), as well as restricted and repetitive patterns of behavior, interests, or activities (e.g., hyper- or hyporeactivity to sensory input or unusual interest in sensory aspects of the environment) (DSM-5 [1]).

Deficits in non-verbal communication and difficulties in imaginative play are associated with imagination. For example, Scott and Baron-Cohen [2] asked children with autism to draw pictures of a real thing (e.g., a house, man, or spider) and something that does not exist (e.g., a house, man, or two-headed monster). Children with autism exhibited a specific impairment in their ability to imagine unusual or unreal objects, which was attributed to possible neural dissociation from other kinds of imagery [2].

Significantly, individuals with ASD exhibit traits such as hyper- or hyporeactivity to sensory input or unusual sensory interest. According to Tavassoli and Miller et al. [3], sensory processing is a key feature of individuals with ASD. Similarly, Kumagai [4] identified hypersensitivity as a core symptom of autism. Takahashi and Masubuchi [5] found that issues of hypersensitivity and insensitivity in people with Asperger’s syndrome and high-functioning autism have not been sufficiently elucidated.

Sensory issues in individuals with and without ASD were investigated using questionnaires measuring sensory features. For example, Crane et al. [6] investigated the Adult/Adolescent Sensory Profile (AASP [7]) and found that sensory abnormalities are prevalent in ASD and that there is considerable within-group variability. Investigating the relationship between the Autism Spectrum Quotient (AQ) [8] and the AASP, Mayer [9] discovered that higher levels of autistic traits are associated with higher levels of sensory function atypicality in both neurotypical and ASD adults. Robertson and Simmons [10] developed the Glasgow Sensory Questionnaire (GSQ) to examine sensory problems and observed positive associations between the GSQ and the AQ, including its subscales. Takayama et al. [11] and Sapey-Triomphe et al. [12] reported similar results. Tavassoli and Miller et al. [3] investigated the relationship between ASD and the Sensory Over-Responsiveness (SensOR) subscale of the Sensory Processing Scale [13]. According to their results, adults with ASD report more sensory over-responsivity than controls across various sensory domains, and SensOR is positively correlated with AQ. Tavassoli and Hoekstra et al. [14] developed the Sensory Perception Quotient (SPQ), a questionnaire that investigates basic sensory functioning. Their findings revealed that adults with ASD report more sensory hypersensitivity to the SPQ than controls, and the SPQ is correlated with the AQ and SensOR. Several studies have indicated that in addition to the total AQ score, each subscale score predicts sensory functioning problems in daily life [9,10,11,12,15].

Imaginative impairment and distinctive sensory characteristics in individuals with ASD should be reflected in their interactions with imagery. However, it remains unclear how these traits are related to imagery traits. Kosslyn [16], based on his model, contended that imagery relies largely on perception mechanisms in the brain because visual images are generated, inspected, maintained, and transformed using the same processing mechanisms of visual perception. Research on the relationship between the AQ and sensory functioning problems allows us to predict the relationship between autistic traits and imagery characteristics.

This study examined the hypothesis that an individual’s degree of “autistic-like traits”, as measured by the AQ, will be reflected in their imagery ability. The AQ assumes cognitive and behavioral continuity among neurotypical individuals, those with Asperger’s syndrome, and those with autism. Specifically, we explored the relationship between autistic-like traits and imagery abilities among college students. Participants were classified into high-, medium-, and low-scoring groups according to the quartile points of the total score and each subscale score of the Japanese version of the AQ [17]; that is, a higher quarter, a medium half, and a lower quarter. According to our prediction, the high-scoring group should exhibit a relatively high degree of autistic-like traits, whereas the low-scoring group should have the least autistic-like traits.

Regarding imagery ability, Hatakeyama classifies it into four dimensions: vividness, controllability, preference (imagery type), and absorption [18]. Large individual differences were revealed long ago by Galton in his study on vividness using the Breakfast Table Questionnaire [19]. Later, Betts developed the Questionnaire upon Mental Imagery (QMI), a 150-item seven-sensory-modality imagery vividness test [20] which Sheehan then shortened to 35 items, 5 for each modality [21]. Marks developed the Vividness of Visual Imagery Questionnaire (VVIQ), a 16-item vision-specific vividness test [22] which is widely used around the world to this day. Marks states: “By ‘vividness’ we mean a combination of clarity and liveliness. The more vivid an image, therefore, the closer it approximates an actual percept.” [23] (p.83). Other tests of imagery ability include Gordon’s Test of Visual Imagery Controllability (TVIC) [24]. This test assesses the ease with which a respondent is capable of imaging car scenes that are presented verbally in succession. Paivio’s Individual Differences Questionnaire (IDQ) [25] and Richardson’s Verbalizer–Visualizer Questionnaire (VVQ) [26] assess preference or imagery type. Imagery type refers to individual differences in imagery of sensory areas that predominate in learning, recall, thinking, and imagining. The IDQ and VVQ focus on whether language or imagery is dominant. Absorption is a dimension of the degree of being absorbed in perceptual stimuli and scenes and fantasies or imaginations. A representative measure is Tellegen and Atkinson’s Absorption Scale (AS) [27]. Although vividness has long been focused on as an imagery ability, it should be noted that vividness is not always necessary for other dimensions, and imagery in other dimensions does not need to be vivid. In this study, three imagery questionnaire tests, the VVIQ, a shortened version of the QMI, and the VVQ, were used to measure imagery ability.

We employed two kinds of analyses. First, to clarify what sort of imagery traits groups exhibit, we compared the scores of three imagery tests between the high-scoring group and the rest of the participants and between the low-scoring group and the other participants. Second, to identify differences between the high- and low-scoring groups, we directly compared the imagery test scores for these two extremes. If the results of the high-scoring group contrast with those of the low-scoring group in the first analysis, there is evidence that an individual’s degree of autistic-like traits affects imagery ability. In such cases, a direct comparison between high- and low-scoring groups in the second analysis should show sharp differences. If only the high-scoring group shows relationships with imagery traits in the first analysis, such imagery traits could be recognized as belonging to individuals with a higher degree of autistic traits. However, if only the low-scoring group shows relationships with imagery traits in the first analysis, such imagery traits could be recognized as belonging to individuals with little or no autistic traits. In these cases, a direct comparison between the two groups does not necessarily reveal differences. In such a way, the first method of analysis can reveal not only the characteristics of the high-scoring group but also those of the low-scoring group.

## 2. Methods

### 2.1. Materials

Autistic-like traits were measured using the Japanese version [17] of the AQ [8], which comprises five subscales: social skill, attention switching, attention to detail, communication, and imagination.

We measured the imagery ability using two vividness tests and one imagery-type test. With respect to the former, we employed the Vividness of Visual Imagery Questionnaire (VVIQ, Marks [22], translated into Japanese by the author), which has four scenes (relative or friend, rising sun, familiar shop, and country scene), and the Questionnaire upon Mental Imagery (QMI, Richardson [28] and Sheehan [21], translated from [28] into Japanese by Onizawa and Takiura), which includes seven modalities (visual, auditory, cutaneous, kinesthetic, gustatory, olfactory, and organic). As an imagery-type test, we used the Verbalizer–Visualizer Questionnaire (VVQ, Richardson [26], translated into Japanese by the author), which consists of both verbal and visual items. However, for the VVQ, we did not adopt Richardson’s [26] original scoring system, which collectively counts positive responses to visual items and negative responses to verbal items as visualizing scores. Instead, we adopted Hatakeyama’s [29] scoring approach, which treats verbalization and visualization scores separately (VVQ-Verbal and VVQ-Visual, respectively).

### 2.2. Participants

The study sample comprised 250 students (125 males and 125 females; 18–23 years old, *M* = 18.9 years) from two colleges in Yamagata and Sendai, Japan. Based on gender, participants were classified into high-, medium-, and low-scoring groups according to the quartile points of the AQ total score and each subscale score (i.e., a higher quarter, a medium half, and a lower quarter). Classification by quartile points is appropriate because the 3rd quartile (75th percentile) point of the AQ total score almost corresponds to the cut-off point for Asperger’s traits [30,31], though cut-off points with respect to the AQ subscales are not known. Participants were classified according to gender based on the finding that males scored higher than females on the AQ total score and subscales of social skill, attention switching, communication, and imagination in Baron-Cohen et al. [8]. Males also scored higher on the AQ total score and on the social skill, communication, and imagination subscales in Wakabayashi et al. [17]. By the classification according to gender, an analysis and discussion can be conducted without considering gender factors. The number of participants differed for each imagery test (VVIQ, *N* = 250; QMI, *N* = 229; VVQ, *N* = 232).

### 2.3. Procedure

The questionnaire survey was conducted in the classroom. On the cover of the questionnaire booklet, the purpose of the study, number of questions, data processing approach, and plan for presentations on meetings were provided. Each participant was required to record the date and their faculty and department, college, year, gender, and age but not their name. Prior to their participation in the study, all participants were informed orally about the purpose of the study, the voluntary nature of participation, anonymity in participation, data processing and research presentation, and the disclosure of information upon request from the participants. Verbal permission was obtained from the participants, and there was no harm. This study was approved by an ethics committee of a college.

### 2.4. Data Analyses

In the first analysis, the scores of the three imagery tests were analyzed using *t*-tests between the high-scoring group and a randomly selected third (25%) of the medium- and low-scoring combined group, and between the low-scoring group and a randomly selected third (25%) of the high- and medium-scoring combined group. The selection of one-third of participants other than the target (experimental) group (i.e., high- or low-scoring groups, respectively) was, for statistical convenience, to ensure that the size of the control group was similar to that of the experimental group. Every third participant was selected according to one of three routines. In the second analysis, direct comparisons were made using *t*-tests between the extremes of the continuum; that is, between the high- and low-scoring groups. 

The second type of analysis, the extreme group approach (EGA), has been widely adopted in studies using the AQ, especially in the field of perception. However, as Preacher et al. [32] point out, there are costs associated with the EGA. An important issue is whether the lower-end group can be an appropriate control group when a large number of medium-scoring individuals are excluded from the spectrum. In this study, the first analysis was more appropriate because the control group comprised all participants except those in the higher- or lower-end groups of a continuum. However, as this was the first exploratory study to investigate the relationship between autistic-like traits and imagery traits, a second analysis comparing both end groups was conducted to elucidate the effect of autistic-like traits on imagery. The first analysis constitutes the primary analysis of this study, and the second is supplementary.

## 3. Results

### 3.1. Basic Statistics of the AQ

The AQ total score averaged 20.48 (*SD* = 6.19), somewhat higher than that of the general control and student groups in Baron-Cohen et al. [8] and similar to that of the general control and student groups in Wakabayashi et al. [17].

The percentage of the cut-off point, 26+ [30,31], was 19.6%, higher than both groups in Baron-Cohen et al. [8] and higher than the general control group and lower than the student group in Wakabayashi et al. [17].

Table 1 shows the correlation coefficients between the AQ total score and each of the subscale scores. The subscale attention to detail was unrelated to the other four subscale scores and had a low correlation with the AQ total score, though significant. So, this subscale does not contribute much to the AQ total score.

### 3.2. Groupings by AQ Score

Table 2 shows the mean AQ total and subscale scores by gender and the classification into high-, middle-, and low-scoring groups by the score, along with range and numbers. For the subscale imagination, males scored significantly higher than females (*t*(248) = 3.05, *p* = 0.003), and for the subscale attention switching, males scored marginally higher than females (*t*(248) = 1.70, *p* = 0.090). No gender differences were seen in the total score or the subscales social skill, attention to detail and communication, which was dissimilar to Baron-Cohen et al. [8] and Wakabayashi et al. [17].

### 3.3. AQ Total Score and Imagery Tests

The results of both analyses are given below: first, the associations of the AQ total score with the imagery tests are provided; subsequently, for each imagery test, the associations of each AQ subscale are shown. As shown in Table 3, Table 4, Table 5 and Table 6, we took note of the results that exhibited statistically significant and also marginally significant differences, revealing how the AQ relates to imagery tests without missing any possible results.

Table 3 exhibits the associations between the AQ total score and the imagery tests.

**Table 3:** Correlation coefficients between the AQ total score and imagery tests and a comparison of the mean scores (*SDs*) of the imagery tests for the groups classified by the AQ total score.

			H vs. M&L				H&M vs. L				H vs. L
	*r*		H	M&L	*p*		H&M	L	*p*		*p*
AQ (Total score)			*N* = 65	*N* = 61			*N* = 60	*N* = 65			
VVIQ (Relative/friend)	0.044		10.25 (3.69)	9.61 (3.42)			10.50 (3.51)	9.66 (3.48)			
VVIQ (Rising sun)	0.099		8.97 (2.86)	8.44 (3.52)			8.43 (3.29)	8.31 (3.57)			
VVIQ (Familiar shop)	−0.083		9.75 (3.63)	10.15 (3.40)			9.92 (3.19)	10.00 (3.41)			
VVIQ (Country scene)	0.020		10.74 (3.40)	10.82 (4.21)			9.92 (3.81)	10.68 (3.55)			
VVIQ (Total score)	0.027		39.71 (9.82)	39.02 (10.87)			38.77 (9.49)	38.65 (10.65)			
AQ (Total score)			*N* = 57	*N* = 57			*N* = 54	*N* = 62			
QMI (Visual)	0.046		12.47 (4.05)	12.36 (4.64)			12.63 (4.76)	12.25 (4.79)			
QMI (Auditory)	0.037		12.63 (4.54)	12.16 (4.41)			11.69 (4.80)	11.98 (4.19)			
QMI (Cutaneous)	0.069		14.25 (5.63)	14.30 (4.89)			13.50 (4.98)	13.15 (4.71)			
QMI (Kinesthetic)	0.078		12.33 (5.27)	11.49 (4.85)			11.63 (5.20)	11.19 (4.46)			
QMI (Gustatory)	0.061		11.46 (4.95)	10.74 (4.42)			10.54 (4.87)	10.77 (4.49)			
QMI (Olfactory)	0.082		15.53 (5.41)	15.05 (6.43)			14.52 (5.18)	*13.85* (5.13)			†
QMI (Organic)	−0.002		10.49 (4.55)	10.70 (5.34)			10.94 (5.35)	10.50 (4.82)			
QMI (Total score)	0.067		88.79 (25.38)	86.75 (26.03)			85.46 (27.31)	83.66 (25.32)			
AQ (Total score)			*N* = 59	*N* = 57			*N* = 54	*N* = 63			
VVQ-Verbal	−0.128 †		2.34 (1.28)	2.65 (1.47)			2.63 (1.63)	*2.83* (1.55)			†
VVQ-Visual	−0.162 *		5.03 (1.76)	**5.72** (1.73)	*		4.98 (1.62)	**5.83** (1.71)	**		*

(Note 1) H: high-scoring group (higher quarter); M: medium-scoring group (medium half); L: low-scoring group (lower quarter). (Note 2) Vividness of Visual Imagery Questionnaire (VVIQ); Questionnaire upon Mental Imagery (QMI): a lower score indicates higher vividness. (Note 3) Verbalizer–Visualizer Questionnaire (VVQ): Verbal = verbalization score; Visual = visualization score. (Note 4) ** *p* < 0.01, * *p* < 0.05, † 0.05 < *p* < 0.10. Mean score in bold—significantly higher characteristic group; in italics—marginally higher characteristic group.

For the VVIQ, the AQ total score did not show any noticeable results in both analyses. For the QMI, in a comparison between both end groups, the high-scoring group (indicating more autistic-like traits) showed marginally weaker olfactory imagery than the low-scoring group (*t*(117) = 1.73, *p* = 0.086). For the VVQ, the AQ total score was related to imagery preference, with the high-scoring group showing significantly lower visualization scores than the medium- and low-scoring combined group (*t*(114) = 2.11, *p* = 0.037), while the low-scoring group (least autistic-like) showed higher visualization scores than the high- and medium-scoring combined group (*t*(115) = 2.73, *p* = 0.007). A direct comparison of the end groups revealed that the high-scoring group had significantly lower visualization scores than the low-scoring group (*t*(120) = 2.52, *p* = 0.013), and moreover, had marginally lower verbalization scores (*t*(120) = 1.88, *p* = 0.062).

### 3.4. AQ Subscales and the VVIQ

Table 4 exhibits the associations between the AQ subscales and the VVIQ.

**Table 4:** Correlation coefficients between the AQ subscales and the VVIQ and a comparison of the mean scores (*SDs*) of the VVIQ for the groups classified by the AQ subscale scores.

			H vs. M&L				H&M vs. L				H vs. L
	*r*		H	M&L	*p*		H&M	L	*p*		*p*
AQ (Social skill)			*N* = 54	*N* = 66			*N* = 63	*N* = 66			
VVIQ (Relative/friend)	0.173 **		10.52 (3.54)	*9.27* (3.43)	†		10.54 (3.41)	**8.94** (3.40)	**		*
VVIQ (Rising sun)	0.105 †		8.94 (3.04)	8.08 (3.34)			9.40 (3.58)	**7.70** (3.59)	**		*
VVIQ (Familiar shop)	−0.035		9.91 (3.37)	9.95 (3.32)			10.78 (3.30)	9.85 (3.50)			
VVIQ (Country scene)	0.037		10.02 (3.42)	10.18 (3.87)			11.43 (3.20)	**9.58** (4.16)	**		
VVIQ (Total score)	0.094		39.39 (9.95)	37.48 (9.91)			42.14 (9.01)	**36.06** (11.93)	***		
AQ (Attention switching)			*N* = 57	*N* = 64			*N* = 62	*N* = 66			
VVIQ (Relative/friend)	0.041		9.51 (3.75)	9.89 (3.48)			9.47 (3.95)	9.59 (3.37)			
VVIQ (Rising sun)	0.048		8.77 (3.15)	8.41 (3.66)			8.05 (3.33)	8.32 (3.73)			
VVIQ (Familiar shop)	−0.044		9.37 (3.58)	10.06 (3.74)			9.34 (3.51)	9.76 (3.24)			
VVIQ (Country scene)	−0.002		10.21 (4.15)	10.02 (3.79)			10.11 (3.93)	10.39 (3.66)			
VVIQ (Total score)	0.014		37.86 (11.04)	38.38 (11.89)			36.97 (11.58)	38.06 (9.27)			
AQ (Attention to detail)			*N* = 59	*N* = 64			*N* = 64	*N* = 56			
VVIQ (Relative/friend)	−0.237 ***		**8.81** (3.48)	10.09 (3.03)	*		**8.56** (3.40)	11.20 (3.18)	***		***
VVIQ (Rising sun)	−0.011		8.41 (3.67)	8.45 (3.22)			**7.63** (2.90)	8.79 (3.25)	*		
VVIQ (Familiar shop)	−0.108 †		***9.53*** (3.50)	10.55 (3.12)	†		10.02 (3.47)	11.04 (3.28)			*
VVIQ (Country scene)	−0.116 †		**9.81** (3.84)	10.66 (3.62)			**9.70** (4.31)	11.27 (3.62)	*		*
VVIQ (Total score)	−0.161 *		***36.56*** (10.26)	39.75 (8.82)	†		**35.91** (11.01)	42.29 (9.99)	***		**
AQ (Communication)			*N* = 47	*N* = 67			*N* = 62	*N* = 64			
VVIQ (Relative/friend)	0.027		9.51 (3.57)	9.28 (3.44)			10.00 (3.95)	9.25 (3.23)			
VVIQ (Rising sun)	0.009		8.23 (2.77)	8.16 (3.65)			8.39 (3.78)	7.86 (3.38)			
VVIQ (Familiar shop)	−0.080		**9.13** (3.41)	10.60 (3.77)	*		10.13 (3.51)	9.64 (3.38)			
VVIQ (Country scene)	0.015		10.21 (3.79)	10.15 (3.97)			10.19 (4.10)	9.86 (3.48)			
VVIQ (Total score)	−0.009		37.09 (9.74)	38.19 (10.96)			38.71 (11.71)	36.61 (9.64)			
AQ (Imagination)			*N* = 61	*N* = 63			*N* = 59	*N* = 71			
VVIQ (Relative/friend)	0.119 †		10.66 (3.61)	**9.27** (3.53)	*		10.75 (3.21)	**9.08** (3.48)	**		*
VVIQ (Rising sun)	0.165 **		9.48 (3.59)	**7.98** (3.66)	*		9.05 (3.56)	*8.25* (3.81)			†
VVIQ (Familiar shop)	0.018		10.41 (3.70)	10.35 (3.31)			11.46 (3.89)	**9.46** (2.93)	***		
VVIQ (Country scene)	0.140 *		11.36 (3.90)	**9.98** (3.71)	*		11.20 (4.22)	***9.89*** (3.58)	†		*
VVIQ (Total score)	0.151 *		41.90 (10.64)	**37.59** (11.03)	*		42.46 (11.27)	**36.69** (9.76)	**		**

(Note 1) H: high-scoring group (higher quarter); M: medium-scoring group (medium half); L: low-scoring group (lower quarter). (Note 2) Vividness of Visual Imagery Questionnaire (VVIQ): a lower score indicates higher vividness. (Note 3) *** *p* < 0.001, ** *p* < 0.01, * *p* < 0.05, † 0.05 < *p* < 0.10. Mean score in bold—significantly higher characteristic group; in italics—marginally higher characteristic group; in italics with bold—marginally higher characteristic group but significantly between both ends.

#### 3.4.1. Social Skill Subscale and the VVIQ

The high-scoring group (unsociable) showed marginally weaker imagery of a scene (i.e., a relative or friend) than the medium- and low-scoring combined group (*t*(118) = 1.95, *p* = 0.053). On the other hand, the low-scoring group (sociable) showed significantly higher vividness of visual imagery than the high- and medium-scoring combined group in three scenes (relative/friend, rising sun, and country) and the total VVIQ score (respectively, *t*(127) = 2.67, *p* = 0.009; *t*(127) = 2.69, *p* = 0.008; *t*(127) = 2.83, *p* = 0.005; *t*(127) = 3.26, *p* = 0.001). Between both end groups, the low-scoring group showed significantly higher vividness of imagery of two scenes (relative/friend and rising sun) (respectively, *t*(118) = 2.49, *p* = 0.014; *t*(118) = 2.03, *p* = 0.045).

#### 3.4.2. Attention-Switching Subscale and the VVIQ

This subscale did not show any noticeable results with the VVIQ.

#### 3.4.3. Attention-to-Detail Subscale and the VVIQ

The high-scoring group (attentive to detail) showed rather marginal but overall higher vividness of visual imagery than the medium- and low-scoring combined group. Specifically, the relative/friend scene showed significance (*t*(121) = 2.18, *p* = 0.031), whereas the familiar shop scene and total VVIQ score showed marginal significance (respectively, *t*(121) = 1.71, *p* = 0.089; *t*(121) = 1.85, *p* = 0.066). In contrast, the low-scoring group (inattentive to detail) showed significantly lower vividness of visual imagery than the high- and medium-scoring combined group in three scenes (relative/friend, rising sun, and country) and total VVIQ score (respectively, *t*(118) = 4.36, *p* = 0.000; *t*(118) = 2.07, *p* = 0.041; *t*(118) = 2.14, *p* = 0.035; *t*(118) = 3.31, *p* = 0.001). When comparing both end groups, the high-scoring group showed significantly higher vividness of visual imagery than the low-scoring group in three scenes (relative/friend, familiar shop, and country) and total VVIQ score (respectively, *t*(113) = 3.83, *p* = 0.000; *t*(113) = 2.39, *p* = 0.019; *t*(113) = 2.09, *p* = 0.039; *t*(113) = 3.03, *p* = 0.003).

#### 3.4.4. Communication Subscale and the VVIQ

The high-scoring group (uncommunicative) showed significantly higher vividness of a visual scene (a familiar shop) than the medium- and low-scoring combined group (*t*(112) = 2.13, *p* = 0.035). For the low-scoring group (communicative), no noticeable results were observed. Between both end groups, no significances were observed.

#### 3.4.5. Imagination Subscale and the VVIQ

The high-scoring and low-scoring groups exhibited close relationships with the vividness of visual imagery. More specifically, the high-scoring group (unimaginative) showed significantly lower imagery vividness than the medium- and low-scoring combined group for the three visual scenes (relative/friend, rising sun, and country) and total VVIQ score (respectively, *t*(122) = 2.16, *p* = 0.032; *t*(122) = 2.29, *p* = 0.024; *t*(122) = 2.02, *p* = 0.046; *t*(122) = 2.22, *p* = 0.029). In contrast, compared to the high- and medium-scoring combined group, the low-scoring group (imaginative) showed significantly higher vividness in the two visual scenes (relative/friend and familiar shop) and total VVIQ score (respectively, *t*(128) = 2.81, *p* = 0.006; *t*(128) = 3.33, *p* = 0.001; *t*(128) = 3.13, *p* = 0.002) and showed marginally higher vividness in a scene (country) (*t*(128) = 1.92, *p* = 0.057). Between both end groups, clear contrasts were seen for imagery vividness, with the high-scoring group (unimaginative) exhibiting significantly lower vividness in the two visual scenes (relative/friend and country) and total VVIQ score (respectively, *t*(130) = 2.54, *p* = 0.012; *t*(130) = 2.26, *p* = 0.025; *t*(130) = 2.93, *p* = 0.004) and marginally lower vividness in a scene (rising sun) (*t*(130) = 1.89, *p* = 0.061).

### 3.5. AQ Subscales and the QMI

Table 5 exhibits the associations between the AQ subscales and the QMI.

#### 3.5.1. Social Skill Subscale and the QMI

The low-scoring group (sociable) showed marginally higher vividness of visual imagery and olfactory imagery (respectively, *t*(118) = 1.73, *p* = 0.086; *t*(118) = 1.74, *p* = 0.084). Between both end groups, the low-scoring group showed significantly higher vividness of olfactory imagery than the high-scoring group (*t*(107) = 2.34, *p* = 0.021).

**Table 5:** Correlation coefficients between the AQ subscales and the QMI and a comparison of the mean scores (*SDs*) of the QMI for the groups classified by the AQ subscale scores.

			H vs. M&L				H&M vs. L				H vs. L
	*r*		H	M&L	*p*		H&M	L	*p*		*p*
AQ (Social skill)			*N* = 47	*N* = 63			*N* = 58	*N* = 62			
QMI (Visual)	0.113 †		12.64 (4.68)	12.31 (4.72)			12.72 (4.28)	*11.34* (4.48)	†		
QMI (Auditory)	0.036		11.43 (4.31)	12.51 (4.07)			12.33 (4.19)	11.50 (4.22)			
QMI (Cutaneous)	0.119 †		13.96 (5.25)	14.22 (4.70)			13.64 (5.14)	12.53 (4.45)			
QMI (Kinesthetic)	0.080		11.72 (5.45)	11.08 (4.36)			11.29 (4.52)	10.71 (4.51)			
QMI (Gustatory)	0.101		11.51 (5.00)	11.61 (4.96)			10.74 (4.75)	10.50 (4.46)			
QMI (Olfactory)	0.152 *		15.94 (5.99)	14.92 (5.18)			15.19 (5.78)	***13.47*** (5.03)	†		*
QMI (Organic)	0.059		9.96 (4.95)	10.92 (4.74)			10.62 (4.22)	9.48 (4.60)			
QMI (Total score)	0.119 †		86.70 (26.38)	88.34 (25.34)			86.43 (25.23)	79.53 (25.13)			
AQ (Attention switching)			*N* = 52	*N* = 59			*N* = 55	*N* = 64			
QMI (Visual)	−0.011		12.00 (4.35)	12.63 (5.13)			11.93 (4.77)	12.53 (4.68)			
QMI (Auditory)	−0.022		11.60 (4.85)	12.02 (4.20)			12.05 (4.80)	12.48 (4.05)			
QMI (Cutaneous)	0.011		13.12 (5.65)	14.20 (5.35)			12.78 (5.15)	13.73 (4.48)			
QMI (Kinesthetic)	0.008		11.29 (4.84)	11.17 (5.15)			10.45 (4.17)	11.47 (4.55)			
QMI (Gustatory)	−0.032		10.62 (4.58)	11.32 (4.70)			10.65 (4.79)	11.34 (4.87)			
QMI (Olfactory)	0.034		14.40 (5.75)	15.46 (6.19)			14.69 (6.09)	14.30 (4.92)			
QMI (Organic)	−0.070		**9.35** (4.13)	11.29 (5.12)	*		10.38 (5.37)	11.22 (4.77)			*
QMI (Total score)	−0.015		82.31 (26.17)	87.83 (29.25)			82.40 (26.13)	87.08 (25.31)			
AQ (Attention to detail)			*N* = 55	*N* = 57			*N* = 59	*N* = 51			
QMI (Visual)	−0.118 †		*11.95* (4.42)	13.39 (4.69)	†		**11.29** (4.57)	13.60 (4.63)	**		†
QMI (Auditory)	−0.018		11.98 (4.58)	12.55 (3.93)			11.75 (4.50)	12.23 (4.79)			
QMI (Cutaneous)	−0.082		13.36 (5.83)	14.00 (4.25)			13.22 (5.02)	14.25 (4.91)			
QMI (Kinesthetic)	−0.067		11.38 (5.27)	12.14 (4.34)			10.95 (4.67)	12.14 (4.74)			
QMI (Gustatory)	−0.059		10.96 (5.49)	11.89 (5.04)			10.57 (4.81)	11.47 (4.24)			
QMI (Olfactory)	−0.092		14.85 (6.47)	14.75 (5.14)			*13.98* (5.23)	15.98 (5.66)	†		
QMI (Organic)	−0.112 †		10.20 (5.37)	11.30 (5.14)			10.00 (4.53)	11.41 (5.31)			
QMI (Total score)	−0.106		84.31 (29.51)	90.29 (24.42)			*82.42* (26.74)	91.08 (25.47)	†		
AQ (Communication)			*N* = 42	*N* = 62			*N* = 60	*N* = 61			
QMI (Visual)	0.063		12.69 (4.46)	11.41 (4.50)			12.07 (4.40)	11.73 (4.69)			
QMI (Auditory)	−0.005		12.10 (4.83)	11.67 (4.49)			11.83 (4.13)	11.90 (4.49)			
QMI (Cutaneous)	0.012		13.50 (5.58)	13.37 (5.54)			13.70 (5.37)	13.15 (4.36)			
QMI (Kinesthetic)	0.058		11.38 (4.56)	11.21 (5.04)			11.02 (4.96)	10.79 (4.33)			
QMI (Gustatory)	0.070		11.15 (4.65)	10.52 (4.77)			10.93 (4.46)	10.44 (4.33)			
QMI (Olfactory)	0.054		14.64 (5.15)	14.58 (6.44)			15.53 (6.17)	*13.72* (5.22)	†		
QMI (Organic)	0.010		10.38 (3.98)	10.29 (5.54)			11.08 (5.47)	9.84 (4.12)			
QMI (Total score)	0.056		86.63 (24.77)	82.90 (28.49)			86.39 (25.65)	81.51 (24.18)			
AQ (Imagination)			*N* = 57	*N* = 60			*N* = 55	*N* = 65			
QMI (Visual)	0.087		13.21 (4.11)	**11.17** (3.95)	**		12.82 (3.79)	12.18 (4.89)			
QMI (Auditory)	0.137 *		13.16 (4.59)	**11.32** (4.38)	*		12.60 (3.86)	**11.62** (4.00)			*
QMI (Cutaneous)	0.159 *		14.60 (4.87)	**12.77** (4.70)	*		15.07 (5.05)	**12.55** (4.47)	**		*
QMI (Kinesthetic)	0.176 **		12.25 (4.89)	11.30 (4.88)			12.95 (5.25)	**10.51** (3.99)	**		*
QMI (Gustatory)	0.103		11.51 (4.78)	10.39 (4.64)			11.89 (4.90)	*10.36* (4.50)	†		
QMI (Olfactory)	0.097		15.46 (4.73)	14.18 (5.85)			16.05 (5.57)	**13.65** (5.12)	*		*
QMI (Organic)	0.106		11.88 (5.06)	**9.90** (4.93)	*		11.36 (4.96)	*10.29* (4.40)			†
QMI (Total score)	0.155 *		91.81 (23.57)	**81.41** (24.38)	*		92.49 (24.96)	**81.52** (24.82)	*		*

(Note 1) H: high-scoring group (higher quarter); M: medium-scoring group (medium half); L: low-scoring group (lower quarter). (Note 2) Questionnaire upon Mental Imagery (QMI): a lower score indicates higher vividness. (Note 3) ** *p* < 0.01, * *p* < 0.05, † 0.05 < *p* < 0.10. Mean score in bold—significantly higher characteristic group; in italics—marginally higher characteristic group; in italics with bold—marginally higher characteristic group but significant between both ends.

#### 3.5.2. Attention-Switching Subscale and the QMI

The high-scoring group (attention-unswitchable) showed significantly higher vividness of organic imagery than the medium- and low-scoring combined group (*t*(109) = 2.18, *p* = 0.031). For the low-scoring group (attention-switchable), no significant differences were observed. In a direct comparison of the two groups, the high-scoring group showed significantly higher vividness of organic imagery than the low-scoring group (*t*(114) = 2.23, *p* = 0.027).

#### 3.5.3. Attention-to-Detail Subscale and the QMI

The high-scoring group (attentive to detail) showed marginally higher vividness of visual imagery than the medium- and low-scoring combined group (*t*(110) = 1.67, *p* = 0.098). In contrast, the low-scoring group (inattentive to detail) showed significantly lower vividness of visual imagery than the high- and medium-scoring combined group (*t*(108) = 2.62, *p* = 0.010), and marginally lower vividness of olfactory imagery, as well as of the whole modality (i.e., the total QMI score) (respectively, *t*(108) = 1.92, *p* = 0.057; *t*(106) = 1.72, *p* = 0.089). When comparing both end groups, the high-scoring group showed marginally higher vividness of visual imagery than the low-scoring group (*t*(105) = 1.89, *p* = 0.062).

#### 3.5.4. Communication Subscale and the QMI

The high-scoring group (uncommunicative) showed no noticeable results. The low-scoring group (communicative) showed marginally more vivid olfactory imagery than the high- and medium-scoring combined group (*t*(119) = 1.74, *p* = 0.084).

#### 3.5.5. Imagination Subscale and the QMI

The high-scoring group (unimaginative) showed significantly lower imagery vividness across the following modalities than the medium- and low-scoring combined group: visual, auditory, cutaneous, and organic imagery and total QMI score (*t*(115) = 2.74, *p* = 0.007; *t*(115) = 2.22, *p* = 0.028; *t*(115) = 2.07, *p* = 0.041; *t*(115) = 2.14, *p* = 0.034; *t*(114) = 2.34, *p* = 0.021). In contrast, compared to the high- and medium-scoring combined group, the low-scoring group (imaginative) showed significantly higher vividness across various modalities: cutaneous, kinesthetic, and olfactory imagery and total QMI score (*t*(118) = 2.90, *p* = 0.004; *t*(118) = 2.89, *p* = 0.005; *t*(118) = 2.47, *p* = 0.015; *t*(117) = 2.40, *p* = 0.018). In addition, the low-scoring group showed marginally higher vividness of gustatory imagery (*t*(117) = 1.78, *p* = 0.078). Between both end groups, clear contrasts were seen for imagery vividness across various modalities, with the high-scoring group (unimaginative) exhibiting significantly lower vividness in auditory, cutaneous, kinesthetic, and olfactory imagery and total QMI score (*t*(120) = 1.98, *p* = 0.050; *t*(120) = 2.42, *p* = 0.017; *t*(120) = 2.16, *p* = 0.033; *t*(120) = 2.02, *p* = 0.046; *t*(119) = 2.33, *p* = 0.021). Additionally, the high-scoring group showed marginally lower vividness in organic imagery (*t*(120) = 1.85, *p* = 0.067). 

### 3.6. AQ Subscales and the VVQ

Table 6 exhibits the associations between the AQ subscales and the VVQ.

**Table 6:** Correlation coefficients between the AQ subscales and the VVQ and a comparison of the mean scores (*SDs*) of the VVQ for the groups classified by the AQ subscale scores.

			H vs. M&L				H&M vs. L				H vs. L
	*r*		H	M&L	*p*		H&M	L	*p*		*p*
AQ (Social skill)			*N* = 49	*N* = 63			*N* = 58	*N* = 60			
VVQ-Verbal	−0.121 †		2.33 (1.55)	2.65 (1.17)			2.53 (1.42)	**2.97** (1.53)			*
VVQ-Visual	−0.202 **		5.06 (1.76)	5.46 (1.76)			5.07 (1.84)	**5.85** (1.64)	*		*
AQ (Attention switching)			*N* = 51	*N* = 60			*N* = 57	*N* = 64			
VVQ-Verbal	−0.083		2.45 (1.32)	2.78 (1.39)			2.60 (1.43)	2.83 (1.45)			
VVQ-Visual	−0.071		5.10 (1.90)	5.42 (1.84)			5.61 (1.66)	5.38 (1.73)			
AQ (Attention to detail)			*N* = 55	*N* = 58			*N* = 59	*N* = 52			
VVQ-Verbal	0.045		2.69 (1.26)	2.43 (1.51)			2.47 (1.25)	2.52 (1.53)			
VVQ-Visual	0.152 *		*5.56* (1.78)	5.05 (1.91)			**5.59** (1.67)	4.94 (1.72)	*		†
AQ (Communication)			*N* = 43	*N* = 63			*N* = 59	*N* = 62			
VVQ-Verbal	−0.132 *		2.40 (1.43)	2.87 (1.49)			2.88 (1.46)	**3.18** (1.52)			**
VVQ-Visual	−0.092		4.93 (1.83)	*5.59* (1.75)	†		5.44 (1.87)	*5.61* (1.73)			†
AQ (Imagination)			*N* = 57	*N* = 59			*N* = 55	*N* = 65			
VVQ-Verbal	−0.105		2.35 (1.43)	**3.05** (1.51)	*		2.51 (1.51)	**3.09** (1.37)	*		**
VVQ-Visual	−0.306 ***		4.51 (1.68)	**5.92** (1.71)	***		5.02 (1.64)	**5.71** (1.67)	*		***

(Note 1) H: high-scoring group (higher quarter); M: medium-scoring group (medium half); L: low-scoring group (lower quarter). (Note 2) Verbalizer–Visualizer Questionnaire (VVQ): Verbal = verbalization score; Visual = visualization score. (Note 3) *** *p* < 0.001, ** *p* < 0.01, * *p* < 0.05, † 0.05 < *p* < 0.10. Mean score in bold—significantly higher characteristic group; in italics—marginally higher characteristic group.

#### 3.6.1. Social Skill Subscale and the VVQ

The high-scoring group (unsociable) did not show any noticeable results. On the other hand, the low-scoring group (sociable) showed significantly more visualization than the high- and medium-scoring combined group (*t*(116) = 2.44, *p* = 0.016). Between both end groups, the low-scoring group showed significantly more verbalization and visualization (respectively, *t*(107) = 2.16, *p* = 0.033; *t*(107) = 2.42, *p* = 0.017).

#### 3.6.2. Attention-Switching Subscale and the VVQ

This subscale did not show any noticeable results with the VVQ.

#### 3.6.3. Attention-to-Detail Subscale and the VVQ

The high-scoring group (attentive to detail) showed no noticeable results. The low-scoring group (inattentive to detail) showed significantly lower visualization than the high- and medium-scoring combined group (*t*(109) = 2.02, *p* = 0.046). When comparing both end groups, the high-scoring group showed marginally higher visualization than the low-scoring group (*t*(105) = 1.83, *p* = 0.070). 

#### 3.6.4. Communication Subscale and the VVQ

The high-scoring group (uncommunicative) showed marginally lower visualization scores than the medium- and low-scoring combined group (*t*(104) = 1.87, *p* = 0.065). The low-scoring group (communicative) did not show any noticeable results. Between both ends, the high-scoring group showed significantly lower verbalization and marginally lower visualization scores than the low-scoring group (respectively, *t*(103) = 2.65, *p* = 0.009; *t*(103) = 1.94, *p* = 0.055).

#### 3.6.5. Imagination Subscale and the VVQ

The high-scoring and low-scoring groups exhibited close relationships between preference. More specifically, the high-scoring group (unimaginative) showed significantly lower verbalization and visualization scores than the medium- and low-scoring combined group (respectively, *t*(114) = 2.56, *p* = 0.012; *t*(114) = 4.47, *p* = 0.000). In contrast, compared to the high- and medium-scoring combined group, the low-scoring group (imaginative) showed higher verbalization and visualization scores (respectively, *t*(118) = 2.22, *p* = 0.029; *t*(118) = 2.27, *p* = 0.025). Between both end groups, clear contrasts were seen for preference. The high-scoring group showed significantly lower verbalization and visualization scores than the low-scoring group (respectively, *t*(120) = 2.92, *p* = 0.004; *t*(120) = 3.94, *p* = 0.000).

### 3.7. Correlation Coefficients between the AQ and Imagery Tests

Concerning correlation coefficients between the AQ and imagery tests, Table 3, Table 4, Table 5 and Table 6 show that coefficients were generally small and, even when statistically significant, most were less than 0.2, with only three coefficients exceeding 0.2. Moreover, discrepancies between the coefficients and our comparative analyses were also revealed. For example, in the case of the AQ imagination subscale, the subscale correlated with VVQ-Visual and did not correlate with VVQ-Verbal, but in the comparisons for both associations, high- and low-scoring groups exhibited respectively clear qualities of visualization and verbalization. Accordingly, we will discuss the results of the comparative analyses in the following section.

## 4. Discussion

ASD is characterized by (1) persistent deficits in social communication and social interaction across multiple contexts and (2) restricted, repetitive patterns of behavior, interests, or activities (DSM-5 [1]). The former are exemplified by symptoms such as deficits in non-verbal communicative behaviors and difficulties in sharing imaginative play; meanwhile, the latter are exemplified by hyper- or hyporeactivity to sensory input or unusual interest in sensory aspects of the environment. Based on the hypothesis that an individual’s degree of autistic-like traits is reflected in their imagery traits, this study examined how the AQ score is related to imagery test scores, particularly observing how imagery traits relate to both characteristics of ASD.

This section discusses the results that exhibited statistically significant and marginally significant differences in the first analysis and those that exhibited statistically significant differences in the second analysis because this study is in the exploratory stage. Doing so is necessary to avoid missing any possible results from the first analysis. Additionally, extreme group comparisons increase the odds of achieving statistical significance (Preacher et al. [32]), allowing for the exclusion of uncertain results in the second analysis.

For the AQ total score, the analyses revealed differences between the high- and low-scoring groups with respect to visualization. Briefly, individuals with a relatively high degree of autistic-like traits as a whole exhibited low visualization scores, whereas those with the fewest autistic traits had high visualization scores. This indicates that a higher degree of possession of autistic-like traits as a whole was related to impediments to visualization but was not related to vividness traits, so we cannot know anything about the vividness dimension for the AQ total score. Why was it not related to the vividness dimension? It is quite probable that setting off the effects of two traits measured by the subscales of the AQ—that is, the merit of “attention to detail” against the fault of “imagination” in imagery qualities, as presented below—resulted in no noticeable differences in the vividness dimension of imagery. If this was the case, the familiar method of classifying participants by their total AQ score would not provide sufficient knowledge of their imagery qualities.

On the AQ, the imagination subscale showed the most extensive relationship with imagery traits. More specifically, the high-scoring group (unimaginative) showed lower imagery vividness across various modalities and lower verbalization and visualization scores, whereas the low-scoring group (imaginative) showed higher imagery traits. A direct comparison revealed a clear contrast between the groups. These findings indicate that imagination is significantly associated with imagery traits. These close relationships may provide important clues for elucidating ASD. Moreover, the results demonstrate that imagination relates not only to imagery vividness and visualization but also to verbalization, suggesting that verbalization is more or less based on imagination. 

We will look over recent studies regarding the effectiveness of the AQ subscales for perceptual characteristics of ASD, anticipating some possible bases for the results. Studies have shown that the imagination subscale of the AQ predicts several issues. Examples include reliance on high-spatial frequency features, that is, detail-focused processing bias (i.e., having an eye for detail) [33]; deficiency in multisensory integration due to a narrow temporal window [34]; and a diminished gaze-orienting effect for happy faces which is attributed to a lack of imagination when explored in experimental conditions using a computer monitor [35]. The first two characteristics, which are typified in detail-focused processing bias, may be the bases for poor imagination, whereas the third is the outcome. 

Following imagination, the attention-to-detail subscale showed a strong relationship with imagery traits. The high-scoring group (attentive to detail) exhibited higher vividness of visual imagery, whereas the low-scoring group (inattentive to detail) showed lower vividness of visual, olfactory, and overall imagery and lower visualization scores. Direct comparisons between the two groups revealed that the attentive-to-detail group had higher vividness of visual imagery. The contrast between the two groups in terms of the vividness of visual imagery indicates that the degree of attention to detail first affects visual imagery vividness. Findings other than visual imagery were solely for the low-scoring group. Lower vividness and visualization are traits of people who are not detail-oriented.

Some studies have suggested that attention to detail plays a role in the vividness of imagery. Marks [36], in his review of historical datasets, showed that detail is one of the key components of visual imagery vividness. Richardson and Patterson [37] reported that multimodal sensory awareness training enhanced imagery vividness wherein participants observed objects minutely and subsequently formed images. Hishitani [38] found that developing expertise increases not only imagery vividness but also the controllability and frequency of imagery use. Skill expertise involves focusing on relevant skills and related events over an extended period. The present study’s finding concerning vividness aligns with those of the aforementioned studies and supports the argument that “attention to the details of a stimulus” acts as a mechanism of imagery vividness (Hatakeyama [18]). ASD sensory traits, such as hyperactivity to sensory input or unusual interest in sensory aspects [1,3,4,5,10], can be categorized as attention to detail.

The attention-to-detail subscale of the AQ has been shown to predict several perceptual issues in ASD, such as superior performance on the Embedded Figure Test (EFT), which is understood as an advantage in processing detail [39], and a diminished gaze-orienting effect for happy faces; that is, a tendency to focus more on details and less on the whole [35]. These characteristics reflect the effects of attention on detailed traits. Alink and Charest [33] reported that among the AQ subscales, only the attention-to-detail subscale was not associated with the reliance-on-detail measure. For individuals with detailed traits, reliance on details may no longer be necessary.

For the social skill subscale, a result revealed a marginal association with the vividness of relative/friend imagery in the high-scoring group (unsociable), indicating that unsociable individuals may have weaker person imagery. Except for this, the social skill subscale exhibited relationships with imagery traits solely in the low-scoring group (sociable), whereby sociable individuals showed higher vividness in visual and olfactory imagery and higher visualization scores. Olfactory imagery may influence sociability. Moreover, a direct comparison between the two groups revealed that the sociable group had also higher verbalization scores than the unsociable group. It is worth noting that the social skill subscale exhibited relationships with imagery traits, almost entirely in the low-scoring group. In other words, these relationships are favorable for sociable individuals.

The social skill subscale of the AQ has been shown to predict several perceptual issues, including superior EFT performance [39,40] and reliance on high-spatial frequency features [33], which are understood as local processing biases. It also predicts impairment in abrupt object discrimination, which is attributed to reduced utilization of the dorsal stream to rapidly activate attention prior to ventral stream processing [41]. Further, it is indicative of a narrow temporal window and deficiency in multisensory integration [34]. It predicts longer evaluation times for unexpected words, which suggests a decreased availability of contextual information [42]. It is notable that all but the last example are perceptual issues, typified by a local processing bias, that cause weak social skills, whereas the last example is the outcome of weak social skills. Regarding imagery traits, the present study found that the AQ social skill subscale was a predictor for the low-scoring (sociable) group but not for the high-scoring (unsociable) group. Although social deficiency is a key symptom of ASD [1], the perceptual mechanisms that would cause social deficits may not be related to imagery qualities.

For the communication subscale, the high-scoring group (uncommunicative) had higher visual imagery vividness of a familiar shop scene but lower visualization and verbalization scores. Although the actual reason for the vividness of familiar shops is unknown, it appears that shops must be special for non-communicative individuals, making their images more vivid. Lower visualization and verbalization scores in an uncommunicative person should be understandable. Meanwhile, the low-scoring group (communicative) showed higher vividness of olfactory imagery. Olfactory imagery could play a role in “communicative” individuals, which is common among “sociable” individuals.

The communication subscale predicted several perceptual issues, including superior EFT performance [39] and reliance on high-spatial-frequency features [33], suggesting a local processing bias and a narrow temporal window [34]. Moreover, the communication subscale of the Autism Diagnostic Interview—Revised (ADI-R [43,44]) showed a negative correlation with an N200 amplitude in coherent motion processing, indicating dorsal stream deficiency [45]. These are unfavorable characteristics for uncommunicative individuals, which may be the basis for their lower visualization and verbalization.

For the attention-switching subscale, the high-scoring (unswitchable) group showed higher vividness of organic imagery in both comparative analyses. This finding suggests that weak attentional switching increases the vividness of organic imagery. Individuals with difficulties in switching attention may pay more attention to organic conditions or responses. The attention-switching subscale has been shown to predict reliance on high-spatial-frequency features, suggesting a local processing bias [33]. This bias may play an important role in weak attention-switching traits and increasing the vividness of organic imagery.

As cited above, it is notable that studies on the effectiveness of AQ subscales for the perceptual characteristics commonly observed, irrespective of varied subscales, a tendency typified by a detail-focused processing bias [33], including a narrow temporal window [34], an advantage in processing detail [39], a tendency to focus more on details [35], a local processing bias [33,34,39,40], and reduced utilization of the dorsal stream [41,45]. Our review here suggests that the “attention to detail” mechanism operates negatively for imagination, social skills, communications, and attention-switching, after the manner of AQ subscales, and would be a core basis of ASD. As for imagery qualities, our results revealed that a local processing bias makes visual images more vivid and makes imagination poorer. 

We summarize the main findings regarding the associations between the AQ and imagery ability. The findings that exhibited contrasts between the high- and low-scoring groups of the AQ are as follows: (1) a higher degree of autistic-like traits as a whole is unfavorable for visualization, while a lower degree is favorable for it; (2) among the AQ subscales, a higher degree of unimaginativeness is unfavorable for imagery vividness across various modalities, visualization, and verbalization, while a lower degree is favorable for them; and (3) a higher degree of attention to detail is favorable for visual imagery vividness, while a lower degree is unfavorable for it. These are evidence that an individual’s degree of autism-like traits affects their imagery traits. 

The findings that did not exhibit contrasts between the high and low groups but did between either group and the other participants are as follows: for the high-scoring group, (1) a higher degree of being unable to switch attention is favorable for organic imagery vividness; and (2) a higher degree of uncommunicativeness is unfavorable for visualization. For the low-scoring group, (3) a lower degree of attention to detail is unfavorable for imagery vividness in visual, olfactory and whole modality, and visualization; (4) a lower degree of unsociableness (i.e., more sociable) is favorable for visual imagery and olfactory imagery vividness, as well as visualization and verbalization; and (5) a lower degree of uncommunicativeness (i.e., more communicative) is favorable for olfactory imagery vividness and verbalization. These indicate that the degree of autistic-like traits affects imagery traits in either of the end groups. 

Table 7 exhibits a summary of the associations between the AQ and imagery ability. It reveals that a higher degree of autistic-like traits is unfavorable for imagery traits or is partly favorable, thereby elucidating how autistic-like traits influence imagery ability. On the other hand, Table 7 also reveals imagery characteristics in individuals with the lowest degree of autistic-like traits. The lowest degree is favorable for imagery traits or partly unfavorable. This shows, on the whole, opposite tendencies of individuals with a higher degree. Synthesizing the results of both degrees, the degree of autistic-like traits is a key factor in imagery ability. In other words, individual differences in autistic-like traits are closely related to individual differences in imagery ability.

With respect to deficits in social communication and social interaction with ASD (DSM-5 [1]), it is worth noting that among the subscales of the AQ, imagination showed the most extensive relationships with imagery traits for both the high-scoring (unimaginative) and low-scoring (imaginative) groups, which contrasted sharply. As for two subscales which involve aspects of sociality, the communication subscale revealed lower visualization scores, though with marginal significance, in the high-scoring (uncommunicative) group, whereas social skill subscale, on the contrary, solely had relationships in the low-scoring (sociable) group. Concerning imagery qualities, these results suggest that imaginative deficits are quite involved in deficits in social communication and interaction in ASD. Symptom exemplifications such as deficits in non-verbal communicative behaviors and difficulties in imaginative play (DSM-5 [1]) are indicative of imagination deficits.

Concerning imagination, Crespi et al. [46] proposed the diametric disorders hypothesis that imagination is reduced in autism and increased in psychotic–affective conditions (schizophrenia, bipolar disorder, and depression). The study first discussed both these two sets of conditions in contrast for nine major aspects and correlates of imagination: (1) pretend play, (2) creativity and generativity, (3) narrative formation and comprehension, (4) mentalizing and empathizing, (5) meaning and salience, (6) episodic memory and future thinking, (7) mental imagery, (8) sensory abilities, and (9) neural activation and connectivity [46] (pp.183-190). Next, it examined the imagination subscale of the AQ and observed that nine of the ten items on this subscale provide a clear measure of brain default mode functions and involve aspects of social imagination [46] (p.190). Here, we can recognize the substances and correlates of imagination and recognize that imagination is specifically reduced in ASD, which the AQ subscale imagination measures optimally. At the same time, in that study, we notice that perceptual bases were referred to in many aspects and correlates of imagination, including reality-based creativity, e.g., [47]; attention focused on non-social aspects [48]; difficulties in switching attention [48]; increased attention to local details, e.g., [49,50,51]; increased focus on predictable patterns [52]; thinking in photorealistic pictures, e.g., [53]; rerunning images on a visuospatial scratch-pad [54]; the increased use of visuospatial processing strategies, e.g., [55]; and increased sensitivities and intense and accurate sensation [3]. Such perceptual bases of imagination in ASD resemble the cases, as discussed above, in which studies on the effectiveness of the AQ subscales for perceptual characteristics commonly observed detail-focused processing biases in all subscales. Baron-Cohen [56], in his systemizing theory of ASD, argued that sensory hypersensitivity is a basis of excellent attention to detail, which is in the service of strong systemizing. These all lead to the views that consider sensory features to be cores of ASD, as seen before [3,4,5,6,9,10,11,12,14,15]. Perceptual bases would be fundamental for imagination in ASD and, first of all, poverty of imagination in ASD would result from a detail-focused processing bias. Crespi et al. [46] appreciate Woodard and Van Reet’s [57] psychological theory of autism based in imagination as highly compatible with previous theories. It proposes developing an imaginative ability which is determined by the degree of progression from part-object/inanimate object to whole-object/human object identification. As such, imagination would be the core phenotype of default mode functions.

According to Harari [58], the Cognitive Revolution occurred between 70,000 and 30,000 years ago, and *Homo sapiens* evolved imagination; that is, the cognitive capacity to believe in fiction. Matsuzawa [59] argued that the capacity to imagine differentiates humans from chimpanzees. Indeed, both a historian and a primate researcher have referred to imagination as a basic trait of humanity. However, individuals with ASD experience difficulties with this trait.

Regarding the distinctive sensory characteristics of ASD, which the DSM-5 [1] exemplifies hyper- or hyporeactivity to sensory input or an unusual interest in sensory aspects for ASD, the attention-to-detail subscale is worth exploring. Our study found that the high-scoring (attentive to detail) group showed higher vividness of visual imagery than the low-scoring (inattentive to detail) group. This finding suggests that a detail-focused processing bias makes visual images more vivid. Furthermore, the finding revealed that in another way, hyperactivity to sensory input creates vividness, whereas hyporeactivity does not. Arguably, in our results, the rather marginal but definite significance for vividness in attentive-to-detail individuals was compounded with more hyperactivity and less hyporeactivity in themselves. If that was the case, it would be essential to discuss the results with statistically marginal significance for the attentive-to-detail group with respect to imagery qualities.

This study provides several suggestions for investigating ASD. First, while research has tended to rely on the total AQ score, utilizing the AQ subscales may prove to be effective. This study derived significant findings and insights using these subscales. Second, to analyze the effects of AQ, this study recommends adopting the methods of comparing the high-scoring group with the rest of the participants (i.e., a randomly selected medium- and low-scoring combined group) and comparing the low-scoring group with the rest of the participants (i.e., a randomly selected high- and medium-scoring combined group). Thinning the participants is carried out to make the size of the control group similar to the target (experimental) group. A direct comparison between the high- and low-scoring groups, that is, the popular extreme group approach, has great problems [32]. Notably, the lower-end group may not necessarily be an appropriate control group because it excludes large number of individuals in the medium-scoring group on a continuum.

## 5. Limitations and Implications of this Study

Limitations regarding the present study should be addressed. First of all, this study used a sample of college students. Their cut-off point for the AQ total score, 26+, was approximately 20%, all of whom were in the high-scoring group, as seen in Table 2. But how our findings may be generalizable is unknown, which is a major limitation. In particular, it is not at all clear how this differs from a sample with more severe ASD traits.

Another is that the cut-off point for the total score, 26, roughly corresponds to the third quartile, so we used quartiles as criterion for classifying participants and applied that approach to the subscales. We assumed that it would be generally appropriate, but it may be unclear whether it is sufficient.

In terms of practical implications, this study may offer the following suggestion. Based on the finding that autistic-like traits are reflected in imagery traits, approaching individuals with ASD from a mental imagery perspective may be helpful. In other words, imagery tests may reveal the characteristics of the inner worlds of individuals with ASD.

## 6. Conclusions

This study is the first to explore relationships between the AQ and imagery tests. The findings indicate the following: (1) the imagery traits shown by the AQ total score are reflected in weak visualization and not in weak vividness. No noticeable differences in the vividness dimension would be attributable to setting off the effects of two traits, i.e., the merit of attention to detail against the demerit of imagination. (2) The imagination subscale shows, most extensively, negative relationships with imagery traits, whereas the communication subscale reveals a negative marginal relationship for visualization; the social skill subscale, on the contrary, is a predictor solely for the low-scoring (sociable) group. Such results suggest that imaginative deficits, in relation to imagery characteristics, would be quite involved in the deficits in social communication and interaction of ASD. (3) The degree of attention to detail primarily affects visual imagery vividness. The rather marginal but definite significance for the high-scoring group would be a compound with more hyperactivity and less hyporeactivity. (4) Given that detail-focused processing bias is a core basis of ASD, the bias makes visual images more vivid and imagination poorer. (5) The results of the lowest degree of autistic-like traits show, on the whole, opposite imagery tendencies to the higher degree. The results indicate that autistic-like traits are associated with qualities of the imagination and especially mental imagery ability.

## Figures and Tables

**Table 1:** Correlation coefficients between AQ total score and subscale scores.

	1	2	3	4	5	6
1 AQ (Total score)	-					
2 AQ (Social skill)	0.731 ***	-				
3 AQ (Attention switching)	0.685 ***	0.361 ***	-			
4 AQ (Attention to detail)	0.317 ***	−0.088	0.098	-		
5 AQ (Communication)	0.774 ***	0.523 ***	0.433 ***	0.064	-	
6 AQ (Imagination)	0.569 ***	0.333 ***	0.286 ***	−0.076	0.322 ***	-

Note: *** *p* < 0.001.

**Table 2:** AQ total score and subscale score by gender and groupings by score.

	Gender						H			M			L	
			*N*	Mean (*SD*)	*p*		Range	*N* (%)		Range	*N* (%)		Range	*N* (%)
AQ (Total score)	Male		125	21.06 (6.42)			25–40	30 (24.0)		17–24	65 (52.0)		4–16	30 (24.0)
	Female		125	19.91 (5.92)			24–38	35 (28.0)		17–23	55 (44.0)		5–16	35 (28.0)
	Total		250	20.48 (6.19)				65 (26.0)			120 (48.0)			65 (26.0)
AQ (Social skill)	Male		125	3.78 (2.35)			6–9	29 (23.2)		3–5	56 (44.8)		0–2	40 (32.0)
	Female		125	3.56 (2.44)			6–10	25 (20.0)		2–5	74 (59.2)		0–1	26 (20.8)
	Total		250	3.67 (2.40)				54 (21.6)			130 (52.0)			66 (26.4)
AQ (Attention switching)	Male		125	5.33 (1.83)	†		7–9	31 (24.8)		5–6	57 (45.6)		0–4	37 (29.6)
	Female		125	4.94 (1.73)			7–9	26 (20.8)		4–6	70 (56.0)		1–3	29 (23.2)
	Total		250	5.14 (1.79)				57 (22.8)			127 (50.8)			66 (26.4)
AQ (Attention to detail)	Male		125	4.93 (2.11)			7–10	32 (25.6)		4–6	59 (47.2)		0–3	34 (27.2)
	Female		125	5.14 (1.87)			7–10	27 (21.6)		4–6	76 (60.8)		1–3	22 (17.6)
	Total		250	5.04 (1.99)				59 (23.6)			135 (54.0)			56 (22.4)
AQ (Communication)	Male		125	3.67 (2.17)			6–10	25 (20.0)		3–5	61 (48.8)		0–2	39 (31.2)
	Female		125	3.56 (2.02)			6–9	22 (17.6)		2–5	78 (62.4)		0–1	25 (20.0)
	Total		250	3.62 (2.09)				47 (18.8)			139 (55.6)			64 (25.6)
AQ (Imagination)	Male		125	3.34 (1.74)	**		5–8	29 (23.2)		3–4	54 (43.2)		0–2	42 (33.6)
	Female		125	2.70 (1.58)			4–9	32 (25.6)		2–3	64 (51.2)		0–1	29 (23.2)
	Total		250	3.02 (1.69)				61 (24.4)			118 (47.2)			71 (28.4)

(Note 1) H: high-scoring group (higher quarter); M: medium-scoring group (medium half); L: low-scoring group (lower quarter). (Note 2) ** *p* < 0.01, † 0.05 < *p* < 0.10.

**Table 7:** Summary of the associations between the AQ and imagery ability.

		High-Scoring Group				Low-Scoring Group		
		Vividness	Verbalization	Visualization		Vividness	Verbalization	Visualization
AQ total score				⚫				⚪
Social skill						⚪ (visual) ○ (olfactory)	⚪	⚪
Attention switching		⚪ (organic)						
Attention to detail		⚪ (visual)				⚫(visual) ● (olfactory) ● (whole)		⚫
Communication				●		○ (olfactory)	⚪	
Imagination		⚫ (various modalities)	⚫	⚫		⚪ (various modalities)	⚪	⚪

(Note) ● unfavorable for imagery ability; ○ favorable for imagery ability; small circle indicates marginal significance.

## Data Availability

The raw data supporting the conclusions of this article will be made available by the author upon request.
